# Acute exposure to simulated nocturnal traffic noise and cardiovascular complications and sleep disturbance—results from a pooled analysis of human field studies

**DOI:** 10.1007/s00392-023-02297-y

**Published:** 2023-09-11

**Authors:** Omar Hahad, Frank P. Schmidt, Jonas Hübner, Patrick Foos, Sadeer Al-Kindi, Volker H. Schmitt, Lukas Hobohm, Karsten Keller, Christina Große-Dresselhaus, Julian Schmeißer, Franziska Koppe-Schmeißer, Markus Vosseler, Donya Gilan, Andreas Schulz, Julian Chalabi, Philipp S. Wild, Andreas Daiber, Johannes Herzog, Thomas Münzel

**Affiliations:** 1 Department of Cardiology, Cardiology I, University Medical Center of the Johannes Gutenberg-University Mainz, Langenbeckstr. 1, 55131 Mainz, Germany; 2 German Center for Cardiovascular Research (DZHK), Partner Site Rhine-Main, Mainz, Germany; 3 Department of Medicine, University Hospitals, Harrington Heart and Vascular Institute, Case Western Reserve University, Cleveland, OH USA; 4 Center for Thrombosis and Hemostasis (CTH), University Medical Center of the Johannes Gutenberg-University Mainz, Mainz, Germany; 5 Department of Sports Medicine, Medical Clinic VII, University Hospital Heidelberg, Heidelberg, Germany; 6 Leibniz Institute for Resilience Research (LIR), Mainz, Germany; 7 Department of Psychiatry and Psychotherapy, University Medical Center of the Johannes Gutenberg-University Mainz, Mainz, Germany; 8 Preventive Cardiology and Preventive Medicine, Department of Cardiology, University Medical Center of the Johannes Gutenberg-University Mainz, Mainz, Germany; 9 Institute for Molecular Biology, Mainz, Germany; 10 Department of Cardiology, Mutterhaus Trier, Trier, Germany

**Keywords:** Environmental risk factor, Traffic noise exposure, Endothelial function, Mean arterial pressure, Sleep disturbance

## Abstract

**Objectives:**

A series of human field studies demonstrated that acute exposure to simulated nocturnal traffic noise is associated with cardiovascular complications and sleep disturbance, including endothelial dysfunction, increased blood pressure, and impaired sleep quality. A pooled analysis of these results remains to be established and is of tremendous interest to consolidate scientific knowledge.

**Methods:**

We analyzed data from four randomized crossover studies (published between 2013 to 2021 and conducted at the University Medical Center Mainz, Germany). A total of 275 subjects (40.4% women, mean age 43.03 years) were each exposed to one control scenario (regular background noise) and at least to one traffic noise scenario (60 aircraft or train noise events) in their homes during nighttime. After each night, the subjects visited the study center for comprehensive cardiovascular function assessment, including the measurement of endothelial function and hemodynamic and biochemical parameters, as well as sleep-related variables.

**Results:**

The pooled analysis revealed a significantly impaired endothelial function when comparing the two different noise sequences (0–60 vs. 60–0 simulated noise events, mean difference in flow-mediated dilation −2.00%, 95% CI −2.32; −1.68, *p* < 0.0001). In concordance, mean arterial pressure was significantly increased after traffic noise exposure (mean difference 2.50 mmHg, 95% CI 0.54; 4.45, *p* = 0.013). Self-reported sleep quality, the restfulness of sleep, and feeling in the morning were significantly impaired after traffic noise exposure (all *p* < 0.0001).

**Discussion:**

Acute exposure to simulated nocturnal traffic noise is associated with endothelial dysfunction, increased mean arterial pressure, and sleep disturbance.

**Supplementary Information:**

The online version contains supplementary material available at 10.1007/s00392-023-02297-y.

## Introduction

Emerging evidence highlights the importance of environmental noise exposure as a substantial public health threat [6, 11]. In support of this, the European Environment Agency concludes that at least about 20% of the EU population in average, in many cities this percentage can reach up to 50% of the urban population, are exposed to long-term noise levels considered potentially harmful to health. Specifically, about 95 million people are exposed to harmful road traffic noise levels. Furthermore, it is estimated that at least 18 million people are highly annoyed and 5 million are highly sleep disturbed by long-term transportation noise in the EU, causing about 11,000 premature deaths and 40,000 new cases of ischemic heart disease per year [1].

During the last few years, strong evidence from epidemiological studies has emerged to demonstrate that traffic noise exposure is a risk factor for cardiovascular disease. For instance, in nationwide studies from Switzerland and Denmark, it was shown that transportation noise exposure is associated with all and cause-specific cardiovascular disease mortality [8, 13, 21] as well as significant cardiovascular outcomes including ischemic heart disease and its’ acute manifestations myocardial infarction, angina pectoris, as well as heart failure, atrial fibrillation, and stroke [18–20]. However, at the same time, it should be noted that evidence from human studies that provide a mechanistic basis for the adverse cardiovascular effects of noise is scarce and scientific knowledge is mainly derived from a series of human field studies [9, 14–16]. In this context, it is important to identify central pathomechanisms underlying the noise-disease-relationship to establish a central framework that displays how noise initiates and contributes to cardiovascular disease. This may help to intensify efforts to officially acknowledge noise not only as an additional noticed enhancer of cardiovascular disease, but as an established and manifest cardiovascular risk factor in a political and medical setting.

In our field studies, we consistently demonstrated that acute exposure to simulated nocturnal aircraft [14–16] or train noise [9] was associated with impaired endothelial function and decreased sleep quality. Less consistent or partly insignificant results were observed in the case of stress hormone release, blood pressure, and other hemodynamic and biochemical parameters. Significant heterogeneity in these studies concerning design and sample included the (a) source of noise—aircraft [14–16] or train noise [9], (b) subjects—younger healthy adults [9, 15] or older subjects with established cardiovascular disease or increased cardiovascular risk [14, 16], (c) number of subjects and sex ratio—*N* = 75 (61% women) [15], *N* = 60 (27% women) [14], *N* = 70 (50% women) [9], or *N* = 70 (20% women) [16], and (d) the number of noise events and corresponding mean sound pressure levels—30 (43 dB(A)) vs. 60 (46 dB(A)) aircraft noise events [15], 60 aircraft noise events (46 dB(A) [14], 30 (52 dB(A)) vs. 60 (54 dB(A)) train noise events [9], or 60 vs. 120 aircraft noise events (both with a mean value of around 45 dB(A)) [16].

To provide overall and robust estimates acknowledging the heterogeneity between studies, we sought to determine the pooled acute effect of simulated nocturnal traffic noise exposure on cardiovascular and sleep-related outcomes based on the data from our human field studies.

## Methods

### Study design

The conception and the design of the included human field studies study have been described in detail previously [9, 14–16]. Briefly, all human field studies were set up as randomized, double-blinded, crossover studies conducted between 2011 and 2020 at the Department of Cardiology of the University Medical Center in Mainz, Germany. Potential participants were found through social media and the distribution of flyers and posters in Mainz, Germany. All participants underwent nights in which they were exposed to a control scenario and at least one noise scenario according to the study protocol. In the morning, after each study night, participants went to the study center for comprehensive and standardized examination follow-up. All participants were exposed to the different scenarios in the participants’ bedrooms in a randomized manner. Study visits were prescheduled with at least three non-study nights between two study nights and on the same weekday, if possible. In female participants, care was taken to synchronize hormonal status with study nights. The control scenario contained no “playback-generated” noise events, but the participants were exposed to normal background noise in their home environments. The noise scenarios consisted of playback of aircraft or train noise events with a varying number of events. The events comprised the noise of a starting or landing aircraft or passing trains. The noise scenarios started with playback of a 30 s lasting tone signaling the beginning of the study night and enabling checking of the equipment. This was followed by 45 min of silence to enable subjects to fall asleep, after which the first noise event was played. Noise events were recorded under controlled circumstances in the bedroom of a resident living near an airport or railway track in Germany. Noise patterns were played back as MP3 files via customary portable audio systems, which were positioned 1 m above the floor at the end of the bed. The sound pressure level (SPL) was continuously measured via class-2 sound level measuring station, which was placed near the head of the participant. Exclusion criteria consisted of the following: exposure to higher residential levels of nocturnal traffic noise as determined by noise maps (L_Aeq,22–6 h_ > 45 dB(A) road traffic, railway, or aircraft noise), being an anti-traffic noise activist, sleep disorder (indicated by a score > 10 on the Pittsburgh Sleep Quality Index (PSQI) [23] or psychiatric disorder assessed by the Mini-International Neuropsychiatric Interview (M.I.N.I.) [17], age-adjusted hearing loss of 30 dB(A) or more, obstructive sleep apnea in the screening test, current shift work and/or regular drug intake except oral contraceptives. Other hormonal therapies led to exclusion. Included participants were advised to refrain from intake of caffeine-containing beverages, alcohol, and supplemental vitamins the day before, during, and in the morning after each study night. Participants were financially compensated when completing the study protocol. All procedures conducted in the field studies were in accordance with the declaration of Helsinki and approved by the Statutory Physician Board of the State Rhineland-Palatinate ethics committee. Written informed consent was obtained before participation in the study. Table 1 gives an overview of the important characteristics of the included studies.

**Table 1:** Characteristics of the included studies

Study, first author (year)	*N*	Source of noise	Age (mean, standard deviation)	Women *N* (%)	Subjects	Study nights, noise scenario, sound pressure levels	Main findings
Schmidt et al. (2013) [15]	75	Aircraft	25.75 ± 6.00	46 (61.3)	Younger healthy adults	Three study nights, control scenario (mean sound pressure and mean maximum levels 35.44 ± 8.08 and 48.63 + 3.47 dB(A), respectively) vs. 30 (43.12 ± 4.91 and 59.89 ± 3.28 dB(A)) vs. 60 (46.28 ± 3.89 and 60.87 ± 2.46 dB(A)) noise events	Noise caused a worsening in sleep quality, a blunting in flow-mediated dilation (FMD), a priming effect (meaning blunting in FMD was particularly evident when subjects were exposed first to 30 and then to 60 noise events), increased adrenaline levels, and increased arterial stiffness (reflected by pulse transit time). Noise-induced endothelial dysfunction was reversed by the administration of vitamin C
Schmidt et al. (2015) [14]	60	Aircraft	61.75 ± 9.21	16 (26.7)	Older subjects with established cardiovascular disease or increased cardiovascular risk	Two study nights, control scenario (mean sound pressure level 39.2 ± 3.1 dB(A)) vs. 60 noise events (46.9 ± 2.0 dB(A))	Subjective sleep quality was markedly reduced by noise, FMD was significantly reduced, and systolic blood pressure was increased by noise. The adverse vascular effects of noise were independent from sleep quality and self-reported noise sensitivity
Herzog et al. (2019) [9]	70	Train	25.74 ± 5.54	35 (50.0)	Younger healthy adults	Three study nights, control scenario (mean sound pressure and mean maximum levels 33.32 ± 4.58 and 64.63 ± 8.62 dB(A), respectively) vs. 30 (52 ± 2.69 and 74.9 ± 3.56 dB(A)) vs. 60 (54.45 ± 2.6 and 74.49 ± 4.02 dB(A)) noise events	FMD was significantly reduced by noise. Sleep quality was impaired after noise exposure. Targeted proteomic analysis showed substantial changes of plasma proteins after the noise night, mainly centered on redox, pro-thrombotic and proinflammatory pathways
Schmidt et al. (2021) [16]	70	Aircraft	62.80 ± 7.06	14 (20.0)	Older subjects with established cardiovascular disease or increased cardiovascular risk	Three study nights, control scenario (mean sound pressure level 36.81 ± 8.34 dB(A)) vs. 60 (44.94 ± 7.49 dB(A)) vs. 120 (45.26 ± 2.78 dB(A)) noise events	FMD and sleep quality were significantly impaired after noise nights. Serial echocardiographic assessment demonstrated an increase in the E/E’ ratio, suggestive of impaired diastolic function

### Examinations

After each study night, participants were invited to the study center in a fasted state, with all measurements conducted and samples collected before 10 a.m. During the study nights, a range of hemodynamic parameters, including heart rate, blood pressure, and pulse transit time, was continuously measured by wearing portable polygraphic screening. The participants put on devices immediately before starting the study night after being carefully advised and trained at the study center. The primary outcome of all studies was endothelial function measured by flow-mediated dilatation (FMD) of the brachial artery using high‐resolution ultrasound based on standardized methods as described [10, 12]. Briefly, the brachial artery diameter was measured before and after an increase in shear stress that is induced by reactive hyperemia. Therefore, the sphygmomanometer cuff was placed proximal to the brachial artery and was then inflated up to 200 mmHg for a period of 5 min. After cuff release, the reactive, flow‐dependent dilation of the brachial artery was recorded. This amount of dilation largely reflects endothelial function. Imaging was performed in a dark, quiet room at a temperature of 21–23 °C. Patients rested in the supine position for at least 10 min before the first scan and remained supine until the final recording was acquired. Image acquisition and analysis were performed under blinded conditions. Measurement of FMD was performed in all studies by a high trained technician with at least 12 months of experience in measuring FMD before getting involved in the endothelial function studies. Afterwards, our in-house clinical chemistry laboratory drew and immediately analyzed blood samples. The sleep quality was evaluated by a visual analog scale (VAS) with the following question: “Overall, how well did you sleep last night?” (VAS ranging from 0 cm meaning very good sleep quality to 10 cm meaning very bad sleep quality). The restfulness of sleep was evaluated with the question: “How restful was your sleep?” (answer format ranging from 0 very good to 5 very bad). Feeling in the morning after the study nights were assessed with the question: “How do you feel now?” (answer format ranging from 0 very good to 15 very bad). Standard laboratory methods were used to determine stress hormone levels and biochemical parameters.

### Statistical analysis

In order to test for a noise exposure effect, within-subject difference in outcome between the two study periods (nights) was calculated and compared between the two different noise sequences (0–60 vs. 60–0 simulated noise events) with an independent samples test. In case of a normal distributed outcome variable, a two-sample *t*-test otherwise a Wilcoxon rank sum test was used. Data were presented as mean/median differences and 95% confidence intervals (CI). To check for potential carry-over effects, the sum of the values measured in the two periods is calculated for each subject and compared across the two sequence groups by means of another test for independent samples. Effect modification by sex and age was analyzed via linear regression with delta as the dependent variable. Moreover, we performed sensitivity analysis by excluding the train noise study [9], as this was the only study that examined train instead of aircraft noise. All tests were two-sided, and *p* values < 0.05 were considered significant. The statistical data analyses were performed using the software R Version 4.2.3 (http://www.r-project.org/).

## Results

### Pooled analysis of the primary outcome—FMD

In total, 275 subjects (111 women, 40.4%) were enrolled with a mean age of 43 ± 20 years.

**Fig. 1:**
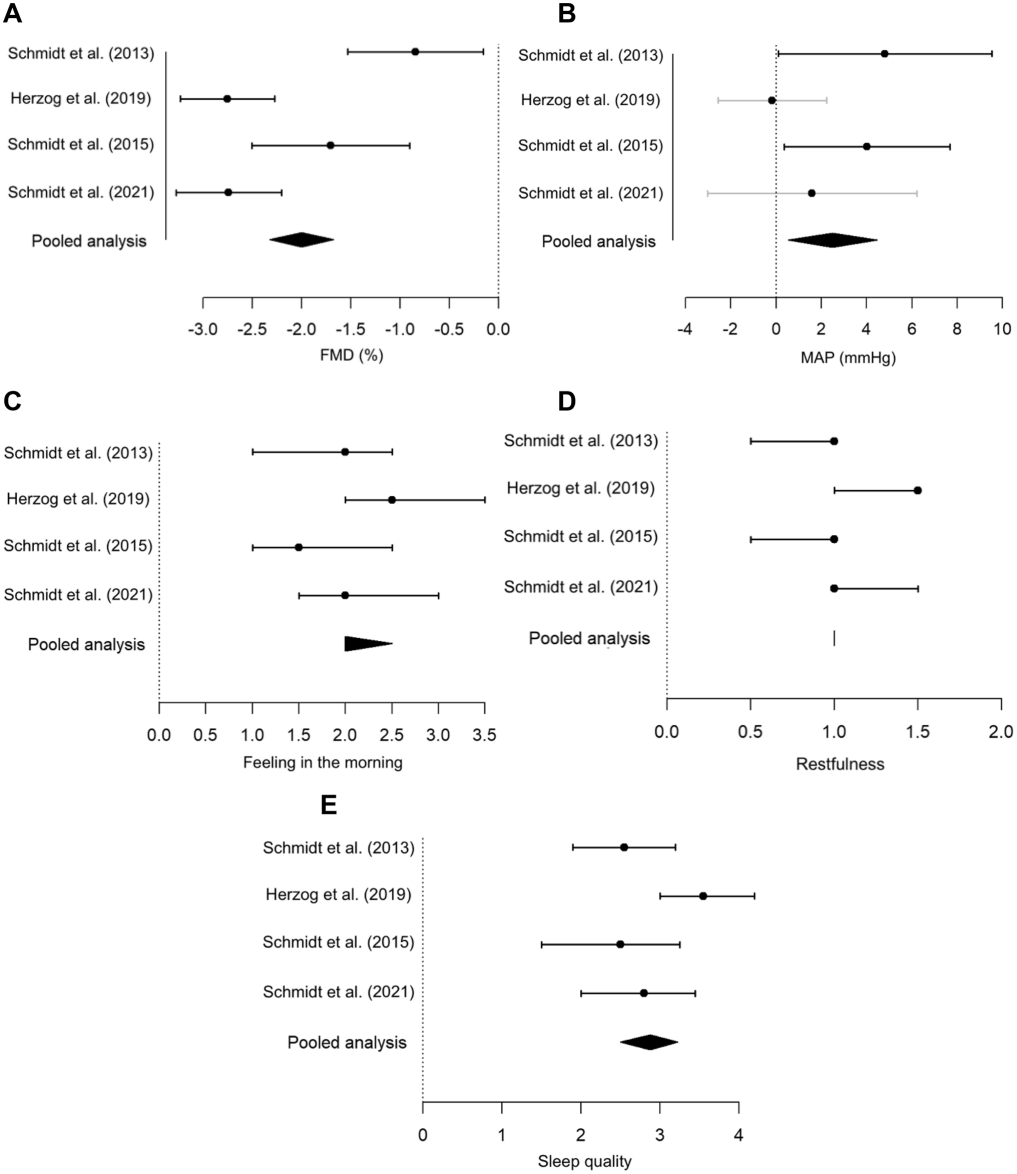
Forest plots of the results presented in the Tables 2, 3, 4, 5 displaying mean or median differences comparing the two different noise sequences (0–60 vs. 60–0 simulated noise events) (*x*-axis) for **A** flow-mediated dilation (FMD %), **B** mean arterial pressure (MAP mmHg), **C** feeling in the morning after study night (answer format ranging from 0 very good to 15 very bad), **D** restfulness (answer format ranging from 0 very good to 5 very bad), and **E** sleep quality (VAS ranging from 0 cm meaning very good sleep quality to 10 cm meaning very bad sleep quality) in our human field studies and pooled results (*y*-axis)

The pooled analysis of the primary outcome, FMD (Table 2 and Fig. 1A), revealed a significant mean difference between the control scenario and noise scenario with 60 events, indicating worsened endothelial function upon noise exposure. Results remained stable after excluding the train noise study [9]. There was no evidence for a carry-over effect. Effect modification analysis indicated no effect of sex and age on the mean difference in FMD (Supplemental Table S1).

**Table 2:** Pooled analysis of the primary outcome—FMD (%)

Study	*N*: 0–60	*N*: 60–0	Mean difference (95% CI)	*p* Value	Test statistic	df	Carry-over effect *p* value
Pooled analysis	134	141	−2.001 (−2.322; −1.681)	** < 0.0001**	12.291	269	0.93
Pooled analysis without Herzog et al. (2019) [9]	100	105	−1.744 (−2.138; −1.35)	** < 0.0001**	8.728	202	0.83
Schmidt et al. (2013) [15]	37	38	−0.847 (−1.536; −0.158)	**0.017**	2.45	71	0.46
Schmidt et al. (2015) [14]	28	32	−1.708 (−2.51; −0.907)	** < 0.0001**	4.289	48	0.17
Herzog et al. (2019) [9]	34	36	−2.755 (−3.235; −2.275	** < 0.0001**	11.474	63	0.87
Schmidt et al. (2021) [16]	35	35	−2.742 (−3.278; −2.206)	** < 0.0001**	10.209	68	0.90

Statistically significant *p* values < 0.05 are in bold

Results were derived from two sample *t*-tests comparing the two different noise sequences (0–60 vs. 60–0 simulated noise events). *FMD* flow-mediated dilation; *CI* confidence interval; *df* degrees of freedom

**Table 3:** Pooled analysis of the secondary outcome—mean arterial pressure (mmHg)

Study	*N*: 0–60	*N*: 60–0	Mean difference (95% CI)	*p* Value	Test statistic	df	Carry-over effect *p* value
Pooled analysis	119	124	2.496 (0.538; 4.453)	**0.013**	−2.512	230	0.17
Pooled analysis without Herzog et al. (2019) [9]	88	93	3.436 (0.955; 5.918)	**0.0070**	−2.734	169	0.14
Schmidt et al. (2013) [15]	28	30	4.814 (0.101; 9.528)	**0.045**	−2.05	52	0.99
Schmidt et al. (2015) [14]	28	31	4.013 (0.359; 7.667)	**0.032**	−2.204	52	0.20
Herzog et al. (2019) [9]	31	31	−0.167 (−2.571; 2.238)	0.89	0.139	60	**0.0056**
Schmidt et al. (2021) [16]	32	32	1.594 (−3.032; 6.22)	0.49	−0.689	61	0.17

Statistically significant *p* values < 0.05 are in bold

Results were derived from two sample *t*-tests comparing the two different noise sequences (0–60 vs. 60–0 simulated noise events). *CI* confidence interval; *df* degrees of freedom

**Table 4:** Pooled analysis of the secondary outcome—feeling in the morning after study night (“How do you feel now?”)

Study	*N*: 0–60	*N*: 60–0	Median difference (95% CI)	*p* Value	Test statistic	Carry-over effect *p* value
Pooled analysis	133	141	2 (2; 2.5)	** < 0.0001**	2423	0.10
Pooled analysis without Herzog et al. (2019) [9]	99	105	2 (1.5; 2.5)	** < 0.0001**	1551	0.20
Schmidt et al. (2013) [15]	36	38	2 (1; 2.5)	** < 0.0001**	225	0.23
Schmidt et al. (2015) [14]	28	32	1.5 (1; 2.5)	** < 0.0001**	142	0.88
Herzog et al. (2019) [9]	34	36	2.5 (2; 3.5)	** < 0.0001**	103.5	0.24
Schmidt et al. (2021) [16]	35	35	2 (1.5; 3)	** < 0.0001**	148	0.29

Statistically significant *p* values < 0.05 are in bold

Results were derived from two sample Wilcoxon rank sum tests comparing the two different noise sequences (0–60 vs. 60–0 simulated noise events). *CI* Confidence interval

**Table 5:** Pooled analysis of the secondary outcome—restfulness (“How restful was your sleep?”)

Study	*N*: 0–60	*N*: 60–0	Median difference (95% CI)	*p* Value	Test statistic	Carry-over effect *p* value
Pooled analysis	133	140	1 [1; 1]	** < 0.0001**	1538.5	**0.012**
Pooled analysis without Herzog et al. (2019) [9]	99	104	1 [1; 1]	** < 0.0001**	1088.5	**0.04**
Schmidt et al. (2013) [15]	36	38	1 [0.5; 1]	** < 0.0001**	207.5	0.059
Schmidt et al. (2015) [14]	28	32	1 [0.5; 1]	** < 0.0001**	100	0.075
Herzog et al. (2019) [9]	34	36	1.5 [1; 1.5]	** < 0.0001**	38.5	0.14
Schmidt et al. (2021) [16]	35	34	1 [1; 1.5]	** < 0.0001**	63	1

Statistically significant *p* values < 0.05 are in bold

Results were derived from two sample Wilcoxon rank sum tests comparing the two different noise sequences (0–60 vs. 60–0 simulated noise events). *CI* Confidence interval

### Pooled analysis of the secondary outcomes

The pooled analysis of secondary outcomes, including mean arterial pressure, feeling in the morning after study night, restfulness, and sleep quality (Tables 3, 4, 5, 6 and Fig. 1B–E) indicated significant mean/median differences, compatible with a higher mean arterial pressure as well as disturbed feeling in the morning, sleep quality, and restfulness due to noise exposure. All estimates remained stable when excluding the train noise study [9]. There was evidence for a carry-over effect in the pooled analysis for restfulness and sleep quality. Significant effect modification by age was revealed in the case of mean differences in the feeling in the morning after study night and sleep quality, indicating a positive association between higher age and an increased disturbed feeling in the morning and sleep quality (Supplemental Tables S2 to S5). No significant mean/median differences were observed in the case of heart rate, the laboratory results regarding adrenaline, cortisol, C-reactive protein, interleukin 6, and neutrophils, as well as pulse transit time (Supplemental Tables S6 to S19).

**Table 6:** Pooled analysis of the secondary outcome—sleep quality (“Overall, how well did you sleep last night?”)

Study	*N*: 0–60	*N*: 60–0	Median difference (95% CI)	*p* Value	Test statistic	Carry-over effect *p* value
Pooled analysis	132	139	2.875 (2.5; 3.225)	** < 0.0001**	1530	**0.0075**
Pooled analysis without Herzog et al. (2019) [9]	98	103	2.6 (2.17; 3.02)	** < 0.0001**	1,082.5	**0.011**
Schmidt et al. (2013) [15]	35	36	2.55 (1.9; 3.2)	** < 0.0001**	81	0.069
Schmidt et al. (2015) [14]	28	32	2.5 (1.5; 3.25)	** < 0.0001**	123	**0.010**
Herzog et al. (2019) [9]	34	36	3.55 (3; 4.2)	** < 0.0001**	28	0.55
Schmidt et al. (2021) [16]	35	35	2.8 (2; 3.45)	** < 0.0001**	134	0.66

Statistically significant *p* values < 0.05 are in bold

Results were derived from two sample Wilcoxon rank sum tests comparing the two different noise sequences (0–60 vs. 60–0 simulated noise events). *CI* confidence interval

## Discussion

The results of our present pooled analysis demonstrate that acute exposure to simulated nocturnal traffic noise is associated with impaired endothelial function, higher mean arterial pressure, and disturbed sleep quality. We further outlined that feeling in the morning and restfulness of sleep were significantly disturbed after noise exposure during night. These results remained stable when excluding the only study in which train instead of aircraft noise was present. While mostly no effect modification by age and sex was observed in primary and secondary outcome variables, differences in sleep quality and feeling in the morning after study night appeared to be modified by age. We found also evidence for a carry-over effect in the pooled analysis for restfulness and sleep quality by applying appropriate statistical methods as recommended for crossover designs in clinical trials [22].

Babisch has proposed a noise reaction model in which the so-called “indirect pathway” is the crucial route by which noise exposure adversely affects the cardiovascular system [2]. Herein, annoyance and interference with daily routines and, importantly, sleep by chronic low-level noise exposure lead to higher psycho-physiological arousal associated with increased stress hormone levels, blood pressure, and heart rate. This, in turn, generates the development and acceleration of cardiovascular risk factors such as arterial hypertension, arrhythmia, dyslipidemia, increased blood viscosity and blood glucose, and activation of blood clotting factors, finally leading to manifest cardiovascular disease over time. In line, we have recently shown that noise annoyance due to different sources is associated with a higher risk of prevalent and incident atrial fibrillation in the Gutenberg Health Study (GHS), including 15,010 participants [3–5]. A further study based on data from the GHS also revealed that noise annoyance due to different sources was associated with increased midregional pro-atrial natriuretic peptide levels, a marker that is associated with endothelial function, which in turn was predictive of incident atrial fibrillation and cardiovascular disease as well as all-cause mortality [7].

Our results are in line with recently published epidemiological studies investigating the relationship between long-term exposure to traffic noise and cardiovascular events, as well as mechanistic animal studies (for review, see [11]). For instance, a nationwide study from Denmark demonstrated that road traffic noise at the most exposed façade was associated with a higher risk of incident ischemic heart disease, myocardial infarction, angina pectoris, and heart failure with hazard ratios (HRs) of 1.052 (95% CI 1.044–1.059), 1.041 (95% CI 1.032–1.051), 1.095 (95% CI 1.071–1.119), and 1.039 (95% CI 1.033–1.045), respectively. Likewise, Vienneau et al. revealed, based on a nationwide cohort from Switzerland, HRs of 1.029 (95% CI 1.024–1.034) and 1.013 (95% CI 1.010–1.017) for the association between road traffic and railway noise and cardiovascular disease mortality, respectively, whereas this association was weaker for aircraft noise (HR 1.003, 95% CI 0.996–1.010) [21]. It is important to note that although these risks increase seem small, the public health impacts are devastating as large parts of the population are routinely exposed to traffic noise and other noise sources [1]. Our results and epidemiological study results suggest that acute reactions in response to traffic noise, such as endothelial dysfunction, increased arterial pressure, and disturbed sleep, will accumulate over time to increase the risk of manifest cardiovascular disease and mortality.

The results of our pooled analysis largely support the noise reaction model, showing important key mechanisms of disease initiation, such as noise-induced endothelial dysfunction, increased arterial pressure, and disturbed sleep. Nevertheless, we did not find evidence of noise-induced changes in heart rate, stress hormones, inflammation, and pulse transit time. Strengths of the present study include the novelty of conducting a pooled analysis of human field studies in the context of acute, controlled exposure to simulated traffic noise, which has several advantages compared to epidemiological designs, where exposure misclassification is more likely and might attenuate/influence or even bias the impact and effect strength of noise on health. A further novel finding includes the positive pooled association between acute nocturnal traffic noise exposure and mean arterial pressure. However, it should be noted that the corresponding confidence intervals are quite wide and that the study from Herzog et al. [9] displays a negative effect estimate, although not significant. Interestingly, this is also the only study in which evidence of a carry-over effect was noticed, which may have interfered with the results. We applied appropriate statistical methods as recommended for crossover designs in clinical trials [22], a substantial advantage compared to the primary studies included in the pooled analysis. Lastly, testing effect modification by sex and age was not done before in this setting. However, the present study also has some limitations that merit consideration. There was evidence for a carry-over effect in the pooled analysis for the secondary outcomes restfulness and sleep quality, which may have interfered with the results. However, care was taken to minimize carry-over effects using counterbalancing (participants were randomly given one of six different sequences of noise and control nights according to the randomization plan) and washout periods (at least three non-study nights between two study nights) where applicable. As stress hormone levels are known to show significant variations over the day, the measurement of associated biomarkers via blood samples hours after awakening may not have been optimal and may explain why stress hormones were not affected by noise in the present analysis. Samples should be collected immediately after awakening by e.g., collecting morning saliva, in future studies. The approach of collecting blood samples directly after awakening may also increase the accuracy of the measurement of other outcomes of interest such as inflammatory markers, that were found not to be affected by noise exposure in the present analysis. However, it may be also the case that an acute scenario of noise exposure is not sufficient to induce a measurable inflammatory response. Measurement of heart rate via standard wearable devices may not be accurate and sensitive enough to detect noise-induced heart rate variations, as it heavily relies on averaging over time periods, as well as it may be susceptible to various disruptive factors during the study night. In addition, measurement of endothelial function in different vascular beds (micro- and macrovascular) would allow a more complete picture of noise-induced vascular damage. Sleep-related variables does not meet the gold standard as it was measured via VAS rather than polysomnography or a wearable device, which could have delivered more objective data on sleep quality. Furthermore, overall sample size is still relatively small, and we cannot rule out the healthy volunteer bias due to the recruitment method. Also, our results only suggest acute noise exposure and not chronic, long-term noise exposure, wherein mechanisms such as adaptation and habituation may come into play. As the present analysis analyzed two distinct samples—young, healthy individuals and older adults with pre-existing cardiovascular conditions, generalizability of the findings to general population is limited, although we did not detect substantial effect modification by age.

In conclusion, our results demonstrate that acute nocturnal traffic noise exposure leads to endothelial dysfunction, higher mean arterial pressure, and disturbed sleep. Our results further highlight key mechanisms regarding the noise-disease-relationship centered on vascular endothelial dysfunction, increased arterial pressure, and impaired sleep quality. Noise is ubiquitous and a major public health challenge, that can only be addressed by appropriate system-level measures.

### Supplementary Information

Below is the link to the electronic supplementary material.

Supplementary file1 (DOCX 176 KB)

## Data Availability

The dataset analyzed for the current study is available from the corresponding author on reasonable request. Applicants will be required to obtain all necessary permissions relevant to data protection regulations before access to data is granted.
