# Publisher Correction: Acute exposure to simulated nocturnal traffic noise and cardiovascular complications and sleep disturbance—results from a pooled analysis of human field studies

**DOI:** 10.1007/s00392-023-02309-x

**Published:** 2023-09-26

**Authors:** Omar Hahad, Frank P. Schmidt, Jonas Hübner, Patrick Foos, Sadeer Al-Kindi, Volker H. Schmitt, Lukas Hobohm, Karsten Keller, Christina Große-Dresselhaus, Julian Schmeißer, Franziska Koppe-Schmeißer, Markus Vosseler, Donya Gilan, Andreas Schulz, Julian Chalabi, Philipp S. Wild, Andreas Daiber, Johannes Herzog, Thomas Münzel

**Affiliations:** 1 Department of Cardiology, Cardiology I, University Medical Center of the Johannes Gutenberg-University Mainz, Langenbeckstr. 1, 55131 Mainz, Germany; 2 German Center for Cardiovascular Research (DZHK), Partner Site Rhine-Main, Mainz, Germany; 3 Department of Medicine, University Hospitals, Harrington Heart and Vascular Institute, Case Western Reserve University, Cleveland, OH USA; 4 Center for Thrombosis and Hemostasis (CTH), University Medical Center of the Johannes Gutenberg-University Mainz, Mainz, Germany; 5 Department of Sports Medicine, Medical Clinic VII, University Hospital Heidelberg, Heidelberg, Germany; 6 Leibniz Institute for Resilience Research (LIR), Mainz, Germany; 7 Department of Psychiatry and Psychotherapy, University Medical Center of the Johannes Gutenberg-University Mainz, Mainz, Germany; 8 Preventive Cardiology and Preventive Medicine, Department of Cardiology, University Medical Center of the Johannes Gutenberg-University Mainz, Mainz, Germany; 9 Institute for Molecular Biology, Mainz, Germany; 10 Department of Cardiology, Mutterhaus Trier, Trier, Germany

**Publisher Correction: Clinical Research in Cardiology** 10.1007/s00392-023-02297-y

By a typesetting mistake, the Table legend of Table 4 was mistakenly added also as last paragraph at the end of the "Pooled analysis of the secondary outcomes" subsection in the "Results" section.

The Original Article has been corrected (and the superfluous paragraph removed).

The publisher apologises for this mistake.

