# Supplementary material for: Acute exposure to simulated nocturnal traffic noise and cardiovascular complications and sleep disturbance—results from a pooled analysis of human field studies
Source: Clin Res Cardiol. 2023 Sep 11;112(11):1690–8. doi: 10.1007/s00392-023-02297-y (PMC10584703; doi:10.1007/s00392-023-02297-y)
Supplement: Supplementary file 1 — Supplementary file1 (DOCX 176 KB) [file 392_2023_2297_MOESM1_ESM.docx]

**Acute exposure to simulated nocturnal traffic noise and cardiovascular complications and sleep disturbance – results from a pooled analysis of human field studies**

Omar Hahad^1,2*^, Frank P. Schmidt^1*^, Jonas Hübner^1^, Patrick Foos^1^, Sadeer Al-Kindi^3^, Volker H. Schmitt^1,2^, Lukas Hobohm^1,4^, Karsten Keller^1,4,5^, Christina Große-Dresselhaus^1^, Julian Schmeißer^1^, Franziska Koppe-Schmeißer^1^, Markus Vosseler^1^, Donya Gilan^6,7^, Andreas Schulz^8^, Julian Chalabi^8^, Philipp S. Wild^8,4,2,9^, Andreas Daiber^1,2^, Johannes Herzog^1^*, Thomas Münzel^1,2^*

*Shared first or senior authorship

From the ^1^ Department of Cardiology, Cardiology I, University Medical Center of the Johannes Gutenberg-University Mainz, Mainz, Germany; ^2^ German Center for Cardiovascular Research (DZHK), Partner Site Rhine-Main, Mainz, Germany; ^3^ University Hospitals, Harrington Heart & Vascular Institute, Department of Medicine, Case Western Reserve University, Cleveland, Ohio, USA ^4^ Center for Thrombosis and Hemostasis (CTH), University Medical Center of the Johannes Gutenberg-University Mainz, Mainz, Germany; ^5^ Medical Clinic VII, Department of Sports Medicine, University Hospital Heidelberg, Heidelberg, Germany; ^6^ Leibniz Institute for Resilience Research (LIR), Mainz, Germany; ^7^ Department of Psychiatry and Psychotherapy, University Medical Center of the Johannes Gutenberg-University Mainz, Mainz, Germany; ^8^ Preventive Cardiology and Preventive Medicine, Department of Cardiology, University Medical Center of the Johannes Gutenberg-University Mainz, Mainz, Germany; ^9^ Institute for Molecular Biology, Mainz, Germany

**^¶^ Address correspondence to:**

Dr. Omar Hahad

University Medical Center at the Johannes Gutenberg University Mainz, Germany

Department of Cardiology, Cardiology I

Langenbeckstr. 1, 55131 Mainz, Germany,

E-mail: [omar.hahad@unimedizin-mainz.de](mailto:omar.hahad@unimedizin-mainz.de)

**Table S1. Effect modification analysis – FMD.**

| Variables | Beta | Lower 95% CI | Upper 95% CI | *P* value |
| --- | --- | --- | --- | --- |
| Intercept | -2.902860 | -4.8487160 | -0.957004 | **0.0042** |
| Sex (women) | 0.002639 | -0.8563551 | 0.861634 | 1.0 |
| Age (years) | 0.059842 | -0.0146541 | 0.134339 | 0.12 |
| Average sound pressure level (dB(A)) (diff.) | -0.100808 | -0.1567177 | -0.044898 | **0.00061** |
| Peak sound pressure level (diff.) | 0.091668 | 0.0358359 | 0.147500 | **0.0017** |
| Body temperature (diff.) | -0.030370 | -0.4066412 | 0.345901 | 0.87 |
| Outdoor temperature (diff.) | -0.049443 | -0.1147008 | 0.015815 | 0.14 |
| Humidity (%) (diff.) | 0.000393 | -0.0000984 | 0.000885 | 0.12 |

Test whether the mean difference (diff.) in flow-mediated dilation (FMD %) between control scenario and noise scenario with 60 events is affected by another (modifier) variable via linear regression analysis. CI: Confidence interval.

**Table S2. Effect modification analysis – mean arterial pressure.**

| Variables | Beta | Lower 95% CI | Upper 95% CI | *P* value |
| --- | --- | --- | --- | --- |
| Intercept | -3.57520 | -17.507 | 10.356 | 0.62 |
| Sex (women) | 4.04013 | -2.299 | 10.380 | 0.22 |
| Age (years) | 0.31467 | -0.216 | 0.845 | 0.25 |
| Average sound pressure level (dB(A)) (diff.) | -0.24010 | -0.640 | 0.159 | 0.24 |
| Peak sound pressure level (diff.) | -0.08509 | -0.509 | 0.339 | 0.70 |
| Body temperature (diff.) | -1.16853 | -3.753 | 1.416 | 0.38 |
| Outdoor temperature (diff.) | -0.25509 | -0.879 | 0.369 | 0.42 |
| Humidity (%) (diff.) | -0.00371 | -0.166 | 0.159 | 0.96 |

Test whether the mean difference (diff.) in mean arterial pressure between control scenario and noise scenario with 60 events is affected by another (modifier) variable via linear regression analysis. CI: Confidence interval.

**Table S3. Effect modification analysis – feeling in the morning after study night (“How do you feel now?”).**

| Variables | Beta | Lower 95% CI | Upper 95% CI | *P* value |
| --- | --- | --- | --- | --- |
| Intercept | -1.235063 | -3.891428 | 1.421302 | 0.36 |
| Sex (women) | 0.734204 | -0.439823 | 1.908230 | 0.22 |
| Age (years) | 0.137598 | 0.035974 | 0.239221 | **0.0092** |
| Average sound pressure level (dB(A)) (diff.) | 0.011783 | -0.064843 | 0.088409 | 0.76 |
| Peak sound pressure level (diff.) | -0.031400 | -0.107559 | 0.044758 | 0.42 |
| Body temperature (diff.) | -0.013466 | -0.526692 | 0.499760 | 0.96 |
| Outdoor temperature (diff.) | 0.105691 | 0.015957 | 0.195426 | 0.023 |
| Humidity (%) (diff.) | -0.000118 | -0.000788 | 0.000553 | 0.73 |

Test whether the mean difference (diff.) in feeling in the morning between control scenario and noise scenario with 60 events is affected by another (modifier) variable via linear regression analysis. CI: Confidence interval.

**Table S4. Effect modification analysis – restfulness (“How restful was your sleep?”).**

| Variables | Beta | Lower 95% CI | Upper 95% CI | *P* value |
| --- | --- | --- | --- | --- |
| Intercept | 0.1039152 | -0.853372 | 1.061202 | 0.83 |
| Sex (women) | 0.0051178 | -0.417972 | 0.428208 | 0.98 |
| Age (years) | 0.0258259 | -0.010797 | 0.062448 | 0.17 |
| Average sound pressure level (dB(A)) (diff.) | 0.0375449 | 0.009931 | 0.065159 | **0.0089** |
| Peak sound pressure level (diff.) | -0.0199016 | -0.047347 | 0.007544 | 0.16 |
| Body temperature (diff.) | 0.1466551 | -0.038299 | 0.331609 | 0.12 |
| Outdoor temperature (diff.) | 0.0179522 | -0.014386 | 0.050290 | 0.28 |
| Humidity (%) (diff.) | 0.0000107 | -0.000231 | 0.000252 | 0.93 |

Test whether the mean difference (diff.) in restfulness between control scenario and noise scenario with 60 events is affected by another (modifier) variable via linear regression analysis. CI: Confidence interval.

**Table S5. Effect modification analysis – sleep quality (“Overall, how well did you sleep last night?“).**

| Variables | Beta | Lower 95% CI | Upper 95% CI | *P* value |
| --- | --- | --- | --- | --- |
| Intercept | -0.8564 | -3.2384 | 1.5256 | 0.48 |
| Sex (women) | -0.2633 | -1.3103 | 0.7837 | 0.62 |
| Age (years) | 0.1742 | 0.0833 | 0.2652 | **0.00029** |
| Average sound pressure level (dB(A)) (diff.) | 0.0227 | -0.0455 | 0.0908 | 0.52 |
| Peak sound pressure level (diff.) | -0.0518 | -0.1197 | 0.0160 | 0.14 |
| Body temperature (diff.) | -0.0186 | -0.4787 | 0.4414 | 0.93 |
| Outdoor temperature (diff.) | 0.0671 | -0.0167 | 0.1508 | 0.12 |
| Humidity (%) (diff.) | -0.0111 | -0.0392 | 0.0171 | 0.44 |

Test whether the mean difference (diff.) in sleep quality between control scenario and noise scenario with 60 events is affected by another (modifier) variable via linear regression analysis. CI: Confidence interval.

**Table S6. Pooled analysis of the secondary outcome – heart rate (bpm).**

| Study | *N*: 0-60 | *N*: 60-0 | Mean difference (95% CI) | *P* value | Test statistic | df | Carry-over effect *p* value |
| --- | --- | --- | --- | --- | --- | --- | --- |
| Pooled analysis | 131 | 132 | 0.392 [-0.213; 0.998] | 0.20318 | -1.276 | 255 | 0.72925 |
| Pooled analysis without Herzog et al. (2019) [1] | 97 | 97 | 0.582 [-0.139; 1.304] | 0.11317 | -1.592 | 187 | 0.84025 |
| Schmidt et al. (2013) [3] | 34 | 32 | 1.173 [-0.225; 2.571] | 0.098556 | -1.677 | 63 | 0.59963 |
| Herzog et al. (2019) [1] | 34 | 35 | -0.138 [-1.264; 0.988] | 0.80771 | 0.244 | 65 | 0.31976 |
| Schmidt et al. (2015) [2] | 28 | 31 | 0.412 [-0.412; 1.236] | 0.32115 | -1.001 | 57 | 0.78883 |
| Schmidt et al. (2021) [4] | 35 | 34 | 0.086 [-1.26; 1.433] | 0.89873 | -0.128 | 65 | 0.74207 |

Results were derived from two sample t-tests comparing the two different noise sequences (0-60 vs. 60-0 simulated noise events). CI: Confidence interval, df: degrees of freedom.

**Table S7. Effect modification analysis – heart rate (bpm).**

| Variables | Beta | Lower 95% CI | Upper 95% CI | *P* value |
| --- | --- | --- | --- | --- |
| Intercept | 0.13145 | -4.5194 | 4.782325 | 0.96 |
| Sex (women) | 0.27771 | -1.7725 | 2.327910 | 0.79 |
| Age (years) | 0.00384 | -0.1730 | 0.180684 | 0.97 |
| Average sound pressure level (dB(A)) (diff.) | -0.04346 | -0.1754 | 0.088432 | 0.52 |
| Peak sound pressure level (diff.) | 0.07670 | -0.0570 | 0.210373 | 0.26 |
| Body temperature (diff.) | -0.79203 | -1.6684 | 0.084388 | 0.080 |
| Outdoor temperature (diff.) | 0.09559 | -0.0707 | 0.261918 | 0.26 |
| Humidity (%) (diff.) | -0.00146 | -0.0026 | -0.000314 | 0.014 |

Test whether the mean difference (diff.) in heart rate between control scenario and noise scenario with 60 events is affected by another (modifier) variable via linear regression analysis. CI: Confidence interval.

**Table S8. Pooled analysis of the secondary outcome – adrenaline (ng/l).**

| Study | *N*: 0-60 | *N*: 60-0 | Median difference (95% CI) | *P* value | Test statistic | Carry-over effect *p* value |
| --- | --- | --- | --- | --- | --- | --- |
| Pooled analysis | 90 | 87 | 0.899 [-0.45; 3.2] | 0.2572 | 3,529.5 | 0.81426 |
| Schmidt et al. (2013) [3] | 37 | 33 | 3.35 [0; 7.6] | 0.053798 | 446.5 | 0.52826 |
| Schmidt et al. (2015) [2] | 26 | 30 | 0 [-5.1; 5.25] | 0.95405 | 386 | 0.71106 |
| Schmidt et al. (2021) [4] | 27 | 24 | 0 [-2.25; 2.45] | 0.83474 | 335.5 | 0.81996 |

Results were derived from two sample Wilcoxon rank sum tests comparing the two different noise sequences (0-60 vs. 60-0 simulated noise events). CI: Confidence interval. Data from [1] was not used as only few values were available.

**Table S9. Effect modification analysis – adrenaline (ng/l).**

| Variables | Beta | Lower 95% CI | Upper 95% CI | *P* value |
| --- | --- | --- | --- | --- |
| Intercept | 12.428179 | -15.17643 | 40.03279 | 0.38 |
| Sex (women) | 5.602806 | -6.40891 | 17.61452 | 0.37 |
| Age (years) | 0.451840 | -0.56545 | 1.46914 | 0.39 |
| Average sound pressure level (dB(A)) (diff.) | -0.392139 | -1.28108 | 0.49680 | 0.393 |
| Peak sound pressure level (diff.) | -1.417187 | -2.64278 | -0.19159 | **0.029** |
| Body temperature (diff.) | 0.859981 | -9.27522 | 10.99518 | 0.87 |
| Outdoor temperature (diff.) | -0.284664 | -1.22076 | 0.65143 | 0.56 |
| Humidity (%) (diff.) | 0.000664 | -0.00376 | 0.00509 | 0.77 |

Test whether the mean difference (diff.) in adrenaline between control scenario and noise scenario with 60 events is affected by another (modifier) variable via linear regression analysis. CI: Confidence interval.

**Table S10. Pooled analysis of the secondary outcome – cortisol (µg/l).**

| Study | *N*: 0-60 | *N*: 60-0 | Mean difference (95% CI) | *P* value | Test statistic | df | Carry-over effect *p* value |
| --- | --- | --- | --- | --- | --- | --- | --- |
| Pooled analysis | 130 | 140 | -0.009 [-0.395; 0.376] | 0.96157 | 0.048 | 268 | 0.32406 |
| Pooled analysis without Herzog et al. (2019) [1] | 96 | 104 | 0.098 [-0.359; 0.554] | 0.67311 | -0.423 | 198 | 0.079087 |
| Schmidt et al. (2013) [3] | 37 | 38 | 0.424 [-0.535; 1.383] | 0.38082 | -0.882 | 73 | 0.14028 |
| Herzog et al. (2019) [1] | 34 | 36 | -0.315 [-1.051; 0.422] | 0.39723 | 0.852 | 68 | 0.30585 |
| Schmidt et al. (2015) [2] | 28 | 32 | -0.498 [-1.293; 0.296] | 0.21412 | 1.256 | 58 | 0.75042 |
| Schmidt et al. (2021) [4] | 31 | 34 | 0.262 [-0.236; 0.76] | 0.29754 | -1.051 | 62 | 0.1465 |

Results were derived from two sample t-tests comparing the two different noise sequences (0-60 vs. 60-0 simulated noise events). CI: Confidence interval, df: degrees of freedom.

**Table S11. Effect modification analysis – cortisol (µg/l).**

| Variables | Beta | Lower 95% CI | Upper 95% CI | *P* value |
| --- | --- | --- | --- | --- |
| Intercept | -0.304112 | -3.378885 | 2.770662 | 0.85 |
| Sex (women) | 0.829799 | -0.527554 | 2.187152 | 0.23 |
| Age (years) | -0.008706 | -0.126423 | 0.109010 | 0.89 |
| Average sound pressure level (dB(A)) (diff.) | -0.004858 | -0.093205 | 0.083489 | 0.91 |
| Peak sound pressure level (diff.) | -0.010424 | -0.098648 | 0.077800 | 0.82 |
| Body temperature (diff.) | -0.062904 | -0.657474 | 0.531667 | 0.83 |
| Outdoor temperature (diff.) | 0.110961 | 0.007842 | 0.214079 | **0.037** |
| Humidity (%) (diff.) | -0.000174 | -0.000951 | 0.000603 | 0.66 |

Test whether the mean difference (diff.) in cortisol between control scenario and noise scenario with 60 events is affected by another (modifier) variable via linear regression analysis. CI: Confidence interval.

**Table S12. Pooled analysis of the secondary outcome – C-reactive protein (mg/l).**

| Study | *N*: 0-60 | *N*: 60-0 | Median difference (95% CI) | *P* value | Test statistic | Carry-over effect *p* value |
| --- | --- | --- | --- | --- | --- | --- |
| Pooled analysis | 131 | 139 | 0.017 [-0.068; 0.107] | 0.67486 | 8835 | 0.68 |
| Pooled analysis without Herzog et al. (2019) [1] | 97 | 103 | 0.021 [-0.072; 0.114] | 0.63708 | 4802 | 0.96393 |
| Schmidt et al. (2013) [3] | 37 | 37 | 0.079 [-0.111; 0.24] | 0.36381 | 600 | 0.66149 |
| Herzog et al. (2019) [1] | 34 | 36 | 0.011 [-0.255; 0.215] | 0.91111 | 602 | 0.44845 |
| Schmidt et al. (2015) [2] | 28 | 32 | -0.03 [-0.185; 0.147] | 0.71856 | 473 | 0.26541 |
| Schmidt et al. (2021) [4] | 32 | 34 | 0.001 [-0.145; 0.16] | 1 | 544 | 0.11018 |

Results were derived from two sample Wilcoxon rank sum tests comparing the two different noise sequences (0-60 vs. 60-0 simulated noise events). CI: Confidence interval.

**Table S13. Effect modification analysis – C-reactive protein (mg/l).**

| Variables | Beta | Lower 95% CI | Upper 95% CI | *P* value |
| --- | --- | --- | --- | --- |
| Intercept | -0.4945417 | -1.647002 | 0.657919 | 0.40 |
| Sex (women) | 0.0215179 | -0.487234 | 0.530269 | 0.93 |
| Age (years) | 0.0052245 | -0.038897 | 0.049346 | 0.82 |
| Average sound pressure level (dB(A)) (diff.) | 0.0190712 | -0.014042 | 0.052185 | 0.26 |
| Peak sound pressure level (diff.) | -0.0013194 | -0.034387 | 0.031748 | 0.94 |
| Body temperature (diff.) | 0.1509156 | -0.071936 | 0.373767 | 0.19 |
| Outdoor temperature (diff.) | -0.0285798 | -0.067230 | 0.010070 | 0.15 |
| Humidity (%) (diff.) | 0.0000152 | -0.000276 | 0.000306 | 0.92 |

Test whether the mean difference (diff.) in C-reactive protein between control scenario and noise scenario with 60 events is affected by another (modifier) variable via linear regression analysis. CI: Confidence interval.

**Table S14. Pooled analysis of the secondary outcome – interleukin 6 (pg/ml).**

| Study | *N*: 0-60 | *N*: 60-0 | Median difference (95% CI) | *P* value | Test statistic | Carry-over effect *p* value |
| --- | --- | --- | --- | --- | --- | --- |
| Pooled analysis | 100 | 104 | 0 [0; 0] | 0.8352 | 5282.5 | 0.54926 |
| Schmidt et al. (2013) [3] | 37 | 38 | 0 [0; 0] | 0.49967 | 745.5 | 0.25384 |
| Schmidt et al. (2015) [2] | 28 | 31 | 0 [-0.024; 0.103] | 0.70907 | 409.5 | 0.4591 |
| Schmidt et al. (2021) [4] | 35 | 35 | 0 [-0.17; 0.109] | 0.73497 | 641.5 | 0.22445 |

Results were derived from two sample Wilcoxon rank sum tests comparing the two different noise sequences (0-60 vs. 60-0 simulated noise events). CI: Confidence interval. Data from [1] was not used as only few values were available.

**Table S15. Effect modification analysis – interleukin 6 (pg/ml).**

| Variables | Beta | Lower 95% CI | Upper 95% CI | *P* value |
| --- | --- | --- | --- | --- |
| Intercept | -0.4945417 | -1.647002 | 0.657919 | 0.40 |
| Sex (women) | 0.0215179 | -0.487234 | 0.530269 | 0.93 |
| Age (years) | 0.0052245 | -0.038897 | 0.049346 | 0.82 |
| Average sound pressure level (dB(A)) (diff.) | 0.0190712 | -0.014042 | 0.052185 | 0.26 |
| Peak sound pressure level (diff.) | -0.0013194 | -0.034387 | 0.031748 | 0.94 |
| Body temperature (diff.) | 0.1509156 | -0.071936 | 0.373767 | 0.19 |
| Outdoor temperature (diff.) | -0.0285798 | -0.067230 | 0.010070 | 0.15 |
| Humidity (%) (diff.) | 0.0000152 | -0.000276 | 0.000306 | 0.92 |

Test whether the mean difference (diff.) in interleukin 6 between control scenario and noise scenario with 60 events is affected by another (modifier) variable via linear regression analysis. CI: Confidence interval.

**Table S16. Pooled analysis of the secondary outcome – neutrophils (%).**

| Study | *N*: 0-60 | *N*: 60-0 | Mean difference (95% CI) | *P* value | Test statistic | df | Carry-over effect *p* value |
| --- | --- | --- | --- | --- | --- | --- | --- |
| Pooled analysis | 131 | 140 | -0.12 [-1.03; 0.79] | 0.79504 | 0.26 | 224 | 0.78878 |
| Pooled analysis without Herzog et al. (2019) [1] | 97 | 104 | -0.487 [-1.551; 0.578] | 0.36791 | 0.903 | 165 | 0.77108 |
| Schmidt et al. (2013) [3] | 37 | 38 | -0.998 [-2.723; 0.727] | 0.25223 | 1.155 | 67 | 0.91066 |
| Herzog et al. (2019) [1] | 34 | 36 | 0.926 [-0.846; 2.698] | 0.29983 | -1.046 | 59 | 0.23944 |
| Schmidt et al. (2015) [2] | 27 | 32 | 0.443 [-1.118; 2.004] | 0.57192 | -0.569 | 56 | 0.25888 |
| Schmidt et al. (2021) [4] | 33 | 34 | -0.654 [-2.825; 1.516] | 0.54582 | 0.609 | 40 | 0.62765 |

Results were derived from two sample t-tests comparing the two different noise sequences (0-60 vs. 60-0 simulated noise events). CI: Confidence interval, df: degrees of freedom.

**Table S17. Effect modification analysis – neutrophils (%).**

| Variables | Beta | Lower 95% CI | Upper 95% CI | *P* value |
| --- | --- | --- | --- | --- |
| Intercept | -1.440283 | -8.10527 | 5.22470 | 0.67 |
| Sex (women) | 0.454754 | -2.48749 | 3.39700 | 0.76 |
| Age (years) | 0.002842 | -0.25232 | 0.25801 | 0.98 |
| Average sound pressure level (dB(A)) (diff.) | 0.065001 | -0.12650 | 0.25650 | 0.51 |
| Peak sound pressure level (diff.) | -0.001420 | -0.19266 | 0.18982 | 0.99 |
| Body temperature (diff.) | 0.537474 | -0.75134 | 1.82629 | 0.42 |
| Outdoor temperature (diff.) | 0.010249 | -0.21327 | 0.23377 | 0.93 |
| Humidity (%) (diff.) | -0.000566 | -0.00225 | 0.00112 | 0.51 |

Test whether the mean difference (diff.) in neutrophils between control scenario and noise scenario with 60 events is affected by another (modifier) variable via linear regression analysis. CI: Confidence interval.

**Table S18. Pooled analysis of the secondary outcome – pulse transit time (m/s).**

| Study | *N*: 0-60 | *N*: 60-0 | Mean difference (95% CI) | *P* value | Test statistic | df | Carry-over effect *p* value |
| --- | --- | --- | --- | --- | --- | --- | --- |
| Pooled analysis | 128 | 130 | 0.334 [-1.125; 1.794] | 0.65222 | -0.451 | 256 | 0.31114 |
| Pooled analysis without Herzog et al. (2019) [1] | 94 | 96 | 0.958 [-0.743; 2.66] | 0.26797 | -1.111 | 187 | 0.044184 |
| Schmidt et al. (2013) [3] | 31 | 31 | -0.871 [-4.091; 2.349] | 0.59021 | 0.542 | 57 | 0.024937 |
| Herzog et al. (2019) [1] | 34 | 34 | -1.441 [-4.202; 1.319] | 0.30104 | 1.042 | 66 | 0.19367 |
| Schmidt et al. (2015) [2] | 28 | 31 | 0.981 [-1.619; 3.581] | 0.45262 | -0.757 | 54 | 0.70075 |
| Schmidt et al. (2021) [4] | 35 | 34 | 2.611 [-0.451; 5.674] | 0.093323 | -1.703 | 64 | 0.18104 |

Results were derived from two sample t-tests comparing the two different noise sequences (0-60 vs. 60-0 simulated noise events). CI: Confidence interval, df: degrees of freedom.

**Table S19. Effect modification analysis – pulse transit time (m/s).**

| Variables | Beta | Lower 95% CI | Upper 95% CI | *P* value |
| --- | --- | --- | --- | --- |
| Intercept | -3.25113 | -14.148610 | 7.64634 | 0.56 |
| Sex (women) | -4.15979 | -8.997471 | 0.67790 | 0.095 |
| Age (years) | 0.15972 | -0.252060 | 0.57151 | 0.45 |
| Average sound pressure level (dB(A)) (diff.) | 0.02146 | -0.286704 | 0.32961 | 0.89 |
| Peak sound pressure level (diff.) | -0.04149 | -0.358601 | 0.27563 | 0.80 |
| Body temperature (diff.) | 0.24823 | -1.770922 | 2.26738 | 0.81 |
| Outdoor temperature (diff.) | -0.16821 | -0.583532 | 0.24711 | 0.43 |
| Humidity (%) (diff.) | 0.00203 | -0.000608 | 0.00466 | 0.14 |

Test whether the mean difference (diff.) in pulse transit time between control scenario and noise scenario with 60 events is affected by another (modifier) variable via linear regression analysis. CI: Confidence interval.

**References**

1. Herzog J, Schmidt FP, Hahad O et al. (2019) Acute exposure to nocturnal train noise induces endothelial dysfunction and pro-thromboinflammatory changes of the plasma proteome in healthy subjects. Basic Res Cardiol 114:46

2. Schmidt F, Kolle K, Kreuder K et al. (2015) Nighttime aircraft noise impairs endothelial function and increases blood pressure in patients with or at high risk for coronary artery disease. Clin Res Cardiol 104:23-30

3. Schmidt FP, Basner M, Kroger G et al. (2013) Effect of nighttime aircraft noise exposure on endothelial function and stress hormone release in healthy adults. Eur Heart J 34:3508-3514a

4. Schmidt FP, Herzog J, Schnorbus B et al. (2021) The impact of aircraft noise on vascular and cardiac function in relation to noise event number: a randomized trial. Cardiovasc Res 117:1382-1390
